# CD105 Expression on CD34-Negative Spindle-Shaped Stromal Cells of Primary Tumor Is an Unfavorable Prognostic Marker in Early Breast Cancer Patients

**DOI:** 10.1371/journal.pone.0121421

**Published:** 2015-03-24

**Authors:** Leandro Marcelo Martinez, Vivian Labovsky, María de Luján Calcagno, Kevin Mauro Davies, Hernán Garcia Rivello, Maria Silvia Bianchi, Alejandra Wernicke, Valeria Beatriz Fernández Vallone, Norma Alejandra Chasseing

**Affiliations:** 1 Immunohematology Laboratory, Experimental Biology and Medicine Institute (IBYME)—National Council of Scientific and Technical Research (CONICET), Ciudad Autónoma de Buenos Aires, Buenos Aires, Argentina; 2 Department of Biostatistics, Faculty of Pharmacy and Biochemistry, University of Buenos Aires, Ciudad Autónoma de Buenos Aires, Buenos Aires, Argentina; 3 Department of Pathological Anatomy, Italian Hospital, Ciudad Autónoma de Buenos Aires, Buenos Aires, Argentina; 4 Neuroendocrinology Laboratory, Experimental Biology and Medicine Institute (IBYME)—National Council of Scientific and Technical Research (CONICET), Ciudad Autónoma de Buenos Aires, Buenos Aires, Argentina; University of Alabama at Birmingham, UNITED STATES

## Abstract

Several studies have confirmed that the breast tumor microenvironment drives cancer progression and metastatic development. The aim of our research was to investigate the prognostic significance of the breast tumor microenvironment in untreated early breast cancer patients. Therefore, we analyzed the association of the expression of α-SMA, FSP, CD105 and CD146 in CD34-negative spindle-shaped stromal cells, not associated with the vasculature, in primary breast tumors with classical prognostic marker levels, metastatic recurrence, local relapse, disease-free survival, metastasis-free survival and the overall survival of patients. In the same way, we evaluated the association of the amount of intra-tumor stroma, fibroblasts, collagen deposition, lymphocytic infiltration and myxoid changes in these samples with the clinical-pathological data previously described. This study is the first to demonstrate the high CD105 expression in this stromal cell type as a possible independent marker of unfavorable prognosis in early breast cancer patients. Our study suggests that this new finding can be useful prognostic marker in the clinical-pathological routine.

## Introduction

Breast cancer (BC) is one of the most common cancers among women worldwide. Statistics indicate that the BC mortality rate in 2000 was 26.7 and 24.2 in Argentina and the United States, respectively; these specific rates are expressed as the number of deaths per year and per 100,000 inhabitants [1], Bulletin N° 96 of Malignant tumor mortality, 1993–1996 and 1997–2000 (2002). National Programme of Health Statistics. Department of Statistics and Health Information, Ministry of Health, Argentina]. Moreover, a study conducted in Argentina between 2008 and 2010 indicated that the highest mortality by cancer in women corresponded to BC, with an annual average of cases of 5,367 per 25,618 (21%) [Bureau of National Statistics and Health Information (2013). Department of Statistics and Health Information, Ministry of Health, Argentina].

In the past decades, many researchers have focused primarily on tumor cells. However, accumulated evidence indicates that tumors are composed of tumor parenchyma and stroma—two discrete but interactive parts that cross-talk to promote tumor growth. Recently, many studies have demonstrated that the breast tumor microenvironment plays an important role in tumor initiation and disease progression [2, 3]. The tumor microenvironment or stroma is formed by cellular and extracellular components [4]. The cellular component includes different types of cells, including myofibroblasts, fibroblasts, myoepithelial cells, blood and lymphatic endothelial cells and their precursors, pericytes, inflammatory cells and mesenchymal stem cells (MSCs) [5]. It is widely recognized that these auxiliary cells collaborate with BC cells to create a tumor-permissive microenvironment capable of providing continuous support for tumor growth, invasion and metastasis [4].

In breast carcinomas, most stromal cells are fibroblasts, and approximately 80% of them are myofibroblasts [3, 6]. These latter cells maintain a spindle shape and have an activated phenotype characterized by increased expression of α-smooth muscle actin (α-SMA) and fibroblast surface protein (FSP), among others markers, and are referred to as cancer-associated fibroblasts (CAFs) [7, 8]. In light of recent findings, CAFs are believed to have 4 major distinct origins: (1) resident mammary fibroblasts [9]; (2) cancer cells that undergo epithelial mesenchymal transition [10]; (3) endothelial cells by endothelial mesenchymal transition [11] and (4) bone marrow MSCs as well as breast tissue-resident MSCs [10, 12]. Independent of the source, CAFs are spindle-shaped stromal cells with negative CD34 expression in invasive breast carcinoma [13, 14]. Other typical markers of MSCs such as CD105 and CD146 could be present in this stromal cell type. Until now, the expression of CD105 and CD146 was described in endothelial progenitors [15–18] and pericytes [19, 20]. Moreover, it was reported that the immunoexpression of microvessels containing CD105 or CD146-labeled endothelial cells is a marker of poor outcome in BC [15–18]. However, the prognostic importance of the expression of α-SMA, FSP, CD105 and CD146 in CD34-negative spindle-shaped stromal cells, not associated with the vasculature, from primary breast tumors remains unknown.

Regarding the above observations, the purpose of our research was to study the prognostic relevance of the tumor microenvironment in a population of BC patients (BCPs) with early-stage disease. Specifically, we analyzed the association of the expression of α-SMA, FSP, CD105 and CD146 in CD34-negative spindle-shaped stromal cells, not associated with the vasculature, in primary breast tumors with classical prognostic marker levels, metastatic recurrence, local relapse, disease-free survival, metastasis-free survival and the overall survival of patients. In the same way, we evaluated the association of the amount of intra-tumor stroma, fibroblasts, collagen deposition, lymphocytic infiltration and myxoid changes in these samples with the clinical-pathological data previously described. In this manuscript, our stromal parameters are called *non-classical prognostic markers*.

## Materials and Methods

This study retrospectively enrolled unselected consecutive cases with a confirmed histological diagnosis made by specialist breast pathologists. In particular, malignant breast tissues obtained by surgical resection from 56 BCPs, as well as non-malignant breast tissue biopsies from 10 control women, were collected. The age of patients ranged between 42 and 80 years, while that for the control women ranged between 45 and 75 years. Key inclusion criteria for BCPs were women with breast infiltrative ductal carcinoma, I and II clinical stages (according to the International Union Against Cancer TNM classification system) and a minimum period of 10 years from the surgery, as well as control women who had negative results of BC. Exclusion criteria were neoadjuvant therapy for BCPs, as well as tissue not available for both groups.

The non-malignant breast tissues used as control were classified as normal breast tissue (n = 6), mammary dysplasia (fibrocystic breast disease, n = 3) and sclerosed fibroadenoma (n = 1).

Paraffin blocks from breast tissues fixed in 10% neutral buffered formalin were retrieved from the surgical archives of the Pathology Department of Italian Hospital, Ciudad de Buenos Aires, Argentina, and then were sectioned at 4-μm thickness for all of the assays. This study and informed consent were approved by the IBYME and Italian Hospital Ethical Committees, and the work was performed in accordance with the principles of the Helsinki Declaration. The informed consent form was signed by each patient or their relatives. The data were anonymous and coded.

After surgery, all of BCPs were treated by specialized Oncologists of Italian Hospital using the indicated hormonal- and/or radio- and/or chemo-therapy depending on their clinical and tumor histopathological characteristics in according to the recommendations of the European Society for Medical Oncology [21].

The cases were collected into a common database. A complete patient record contained information concerning the following clinicopathological characteristics: age, tumor size, histological grade, status of HER2/neu, hormonal receptors [estrogen receptors (ER) and progesterone receptors (PR)] and regional lymph nodes (*classical prognostic markers*), metastatic recurrence, local relapse, disease-free survival, metastasis-free survival and overall survival.

Analysis of the classical prognostic markers were a priority determined according to Italian Hospital´s protocols as described Wernicke M et al. [22]. Specifically: *a)* we subdivided our population into two age groups: < 50 and ≥ 50 years old; *b)* the tumor size was subdivided into < 2 and ≥ 2 cm; *c)* histological grade was evaluated based on the Scarff-Bloom-Richardson grading system [23] and was categorized as well differentiated/intermediate (G1/G2) and poor (G3); *d)* hormonal receptor and HER2/neu expression was considered to be negative or positive according to Wernicke M et al. [22]; and *e)* the presence of regional metastatic lymph nodes was recorded as negative (negative nodes in axillary dissection or negative sentinel lymph node when this technique was performed) or positive (including micrometastasis) (Table 1).

**Table 1:** Clinicopathological characteristics of 56 untreated early breast cancer patients.

*Characteristics*	*Patients (n)*	*Patients (%)*
***Age (years)***		
*< 50*	8	14.28
*≥ 50*	43	76.79
*Unknown*	5	8.93
***Tumor size (cm)***		
*<2*	41	73.21
*≥ 2*	14	25.00
*Unknown*	1	1.79
***Histological grade***		
*G1/G2*	32	57.14
*G3*	23	41.07
*Unknown*	1	1.79
***HER2/neu status***		
*Negative*	32	57.14
*Positive*	23	41.07
*Unknown*	1	1.79
***ER status***		
*Negative*	15	26.79
*Positive*	40	71.43
*Unknown*	1	1.79
***PR status***		
*Negative*	14	25.00
*Positive*	41	73.21
*Unknown*	1	1.79
***Regional lymph nodes***		
*Negative*	40	71.43
*Positive*	14	25.00
*Unknown*	2	3.57
***Metastatic recurrence***		
*Negative*	39	69.64
*Positive*	11	19.64
*Unknown*	6	10.71
***Sites of metastasis***		
*No metastasis*	39	69.64
*Bone*	5	8.93
*Viscera and non-regional lymph nodes*	3	5.36
*Bone + viscera and non-regional lymph nodes*	3	5.36
*Unknown*	6	10.71
***Local relapse***		
*Negative*	45	80.36
*Positive*	5	8.93
*Unknown*	6	10.71

HER2: Human epidermal growth factor receptor 2; ER: Estrogen receptor; PR: Progesterone receptor

### Immunohistochemical assay

Sections were deparaffinized and hydrated to passages in xylene and 100, 96 and 70% ethanol and then were incubated in citrate buffer (0.01 M, pH 6) at 60°C for 25 min. Endogenous peroxidase was blocked by incubating the tissues with 3% hydrogen peroxide for 5 min. Next, proteins were blocked using 1% bovine serum albumin in phosphate-buffered saline-0.1% Tween-20 for 1 h. Thereafter, the tissues were incubated with the following primary human antibodies (Abs): anti- α-SMA (mouse IgG2a; MAB1420; R&D Systems), FSP (mouse IgM; F4771; Sigma-Aldrich), CD105 (goat IgG; AF1097; R&D Systems) or CD146 (goat IgG; AF932; R&D Systems) overnight at 4°C in a humid environment. Next, LSAB+ System-HRP (K0690; Dako) and 3-3´-diaminobenzidine (Liquid DAB+ Substrate Chromogen System; K3468; Dako) were used according to the manufacturer’s specifications. Hematoxylin (Biopur) was used for counterstaining, followed by mounting with Canada Balsam (Canadax, Biopur). Tissue sections were also incubated without primary Abs and with irrelevant mouse IgG2a (X0943; Dako), irrelevant mouse IgM (X0942; Dako) or total normal goat IgG (AB-108-C; R&D Systems) as negative controls in all of the runs. Each sample was assayed in duplicate.

The immunohistochemical reactions were estimated independently by two pathologists. The agreement in immunohistochemical evaluation between the two observers was 85.58% (Kappa value: 0.8175). In cases with no agreement, a re-evaluation was performed using a double-headed microscope, and staining was discussed until a consensus was achieved. Each slide was initially examined by these pathologists at 100× magnification for an overall view, and then 5 representative fields at 400× magnification were systematically evaluated. The immunohistochemical signal was scored based on the method by Surowiak P et al. [24]. Briefly, a quantitative score was assigned representing the percentage of stromal cells with positive staining [0% (score 0); <10% (score 1); 10–50% (score 2); 51–80% (score 3) or >80% (score 4)]. The intensity of staining in positive cells was assigned an intensity score [absent (score 0); low (score 1); moderate (score 2) or intense (score 3) by comparison with internal controls]. The quantitative score and intensity score were added to obtain a total score that ranged from 0 to 7. The intensity score was based on the relative intensities of stromal cell staining in reference to the staining present in specific cells of breast tissue. As previously described, breast tissue demonstrated the immunoreactivity of α-SMA on smooth muscle cells and myoepithelial cells [25], FSP on tissue macrophages [26] and CD105 and CD146 on endothelial cells [15–18]. These internal references were used as internal positive controls.

Although it is known that the spindle-shaped stromal cells have negative CD34 expression in invasive breast carcinoma [13, 14], we analyzed its expression in the evaluated stromal cells to confirm that we did not consider individual endothelial progenitor cells in malignant tissues. Specimens were incubated with Ab anti-CD34 (mouse IgG1; M7165; Dako) or irrelevant mouse IgG1 (MAB002; R&D Systems) as a negative isotype control in all of the runs. Each sample was also assayed in duplicate. Moreover, we also performed two-color immunohistochemistry for CD105 and CD34. The double immunostaining involved sequential staining of each primary Ab anti-CD105 (detected by LSAB+ System-HRP and 3-3´-diaminobenzidine as previously described) and CD34 [detected by Biotinylated anti-mouse IgG (H+L; BA-2000; Vector Laboratories), Vectastain ABC-Alkaline Phosphatase kit (Ak-5000; Vector Laboratories) and Vector Red Substrate Kit (SK-5100; Vector Laboratories) according to the manufacturer’s specifications]. Nuclei were counterstained with hematoxylin followed by mounting with VectaMount mounting medium (H-5000; Vector Laboratories). Tissue sections were also sequentially incubated with total normal goat IgG and irrelevant mouse IgG1 as the negative control in all of the runs. Each sample was also assayed in duplicate. In parallel, we also performed single immunostaining for CD34 using red chromogen in these tissue sections as previously described. Tissue sections were also incubated with irrelevant mouse IgG1 as a negative isotype control in all of the runs. Each sample was also assayed in duplicate.

### Hematoxylin and Eosin (H&E) staining

The study of *tumor stromal histological features* as intra-tumor stroma and amount of fibroblasts, collagen deposition, lymphocytic infiltration and myxoid changes were determined by routine H&E staining. First, using a 5× objective, the most invasive tumor area of the whole tissue slide was selected. Subsequently, using a 10× objective, only fields were evaluated where both stroma and tumor were present as described Kruijf EM et al. [27]. Specifically, the stromal myxoid changes were evaluated based on Wernicke M et al. [28]. The slides were estimated independently by two pathologists as previously described. The amount of fibroblasts, collagen deposition, lymphocytic infiltration and myxoid changes was scored as absent (score 0); scanty (score 1); moderate (score 2) or large amount (score 3) by these pathologists. The agreement in the evaluation of these stromal features between the two observers was 89.42% (Kappa value: 0.8455). By contrast, the intra-tumor stroma amount was expressed as a percentage. In this case, the intraclass correlation coefficient (ICC) to estimated the observer variation was 0.8967. Each sample was assayed in duplicate.

### Statistical analysis

The difference in stromal marker expression between breast malignant tissues and non-malignant tissues was analyzed by the Mann-Whitney test.

To evaluate the associations between non-classical prognostic markers and clinicopathological characteristics in BCPs, we identified a cut-off value according to the results of previous studies [29, 30]. Thus, the cut-off value (≤ or >) was used for binomial classification of tumor samples: negative/low or high expression, as well as negative or positive expression as appropriate (stromal cell markers), absent/scanty or large amount (fibroblasts, collagen deposition, lymphocytic infiltration and myxoid changes) and ≤ or > percentage (intra-tumor stroma). To identify the optimal cut-off value, Q1, median and Q3 values were tested individually in univariate analysis with respect to overall survival of the BCPs. The cut-off value that showed the lowest p-value was chosen. The optimal cut-off values of the expression of α-SMA, FSP, CD105 and CD146, as well as those for the amount of intra-tumor stroma, fibroblasts, collagen deposition, lymphocytic infiltration and myxoid changes were as follows: 5 (Q1), 0 (Q1), 3 (Q3), 0 (median or Q1), 50% (median), 1 (median or Q1), 1 (Q1), 1 (Q1, median, Q3) and 1 (Q1, median, Q3), respectively. Consequently, the association of non-classical prognostic markers with classical prognostic markers, metastatic recurrence and local relapse was analyzed using Fisher’s exact test.

Disease-free survival and metastasis-free survival were calculated as the period from the date of surgery to the first observation of tumor recurrence (metastatic recurrence and/or local relapse) and metastatic recurrence, respectively, or last follow-up. The overall survival was calculated as the period from the date of surgery until death from the BC cause or last follow-up. Univariate analysis of disease-free survival, metastasis-free survival and overall survival was estimated according to the Kaplan-Meier method and analyzed by the log-rank (Mantel-Cox) test. Once significant variables (classical and non-classical prognostic markers) were identified, we applied the Cox proportional hazards model to the multivariate survival analysis incorporating only these significant variables.

Multiple testing was corrected using Benjamini-Hochberg false discovery rate [31].

A p-value <0.05 was considered to be statistically significant (two sided p-value). Statistical analysis was performed by an expert statistician using SPSS software (version 18.00, Chicago, Illinois) and InfoStat (version 2012, InfoStat Group, National University of Cordoba, Argentina).

## Results

### Expression of α-SMA, FSP, CD105 and CD146 in spindle-shaped stromal cells, not associated with the vasculature, in malignant and non-malignant breast tissues

**Fig 1:**
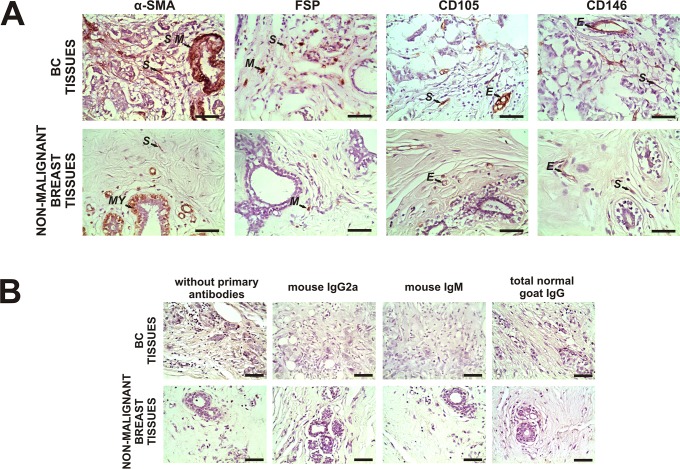
Expression of α-SMA, FSP, CD105 and CD146 in malignant and non-malignant breast tissues. **A** Representative immunohistochemistry staining for α-SMA, FSP, CD105 and CD146 in stromal cells of primary tumor tissues from breast cancer (BC) patients and non-malignant breast tissues. Reactions were evaluated in spindle-shaped stromal cells, not associated with the vasculature. The arrows show positive staining of evaluated stromal cells (S) and smooth muscle cells (S M), myoepithelial cells (MY), tissue-associated macrophages (M) and endothelial cells (E) used as internal positive controls as previously described. **B** No staining was observed in both types of tissues when we incubated them without primary antibodies and with irrelevant mouse IgG2a (for α-SMA), irrelevant mouse IgM (for FSP) or total normal goat IgG (for CD105 and CD146) as negative controls. Nuclei were counterstained with hematoxylin (purple). Original magnification A and B: 400×. The scale bars represent 50 μm.

BC tissues (n = 54) exhibited significantly higher stromal cell expression of α-SMA than non-malignant breast tissues (n = 9) [data expressed as median (interquartile range), Mann-Whitney test]: 6 (5–7) vs. 4 (0–4), respectively (p = 0.0001; Benjamini-Hochberg adjusted p = 0.0065). In addition, stromal cells of BC tissue expressed FSP (n = 53) and CD105 (n = 52) [data expressed as median (interquartile range)]: 2 (0–3) in both cases, but there was no expression of these markers in non-malignant breast tissue (n = 8 and 6, respectively). By contrast, data showed expression of CD146 in both types of tissues, but no significant difference was found [n = 56 (BC samples) and n = 10 (non-malignant samples)] (Fig. 1).

### Expression of CD34, as well as that of CD105 and CD34, in spindle-shaped stromal cells, not associated with the vasculature, in malignant breast tissues

**Fig 2:**
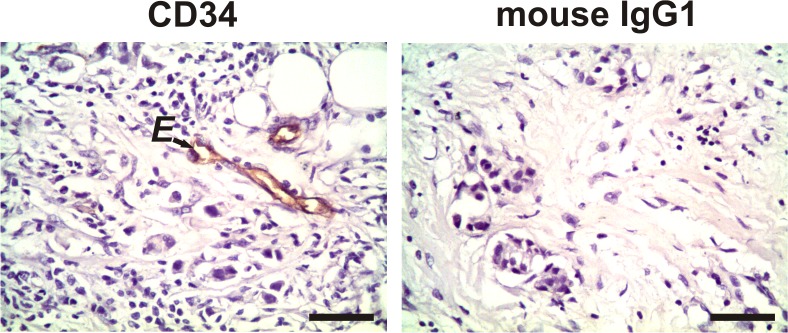
Expression of CD34 in tumor stromal cells. Representative negative staining for CD34 in the evaluated spindle-shaped stromal cells, not associated with the vasculature, of primary tumor tissue from a breast cancer patient. The arrow shows an example of CD34-positive staining only in the endothelial cells (E). No staining was observed in the tissues when we incubated them with irrelevant mouse IgG1 as a negative isotype control. Nuclei were counterstained with hematoxylin (purple). Original magnification: 400×. The scale bar represents 50 μm.

**Fig 3:**
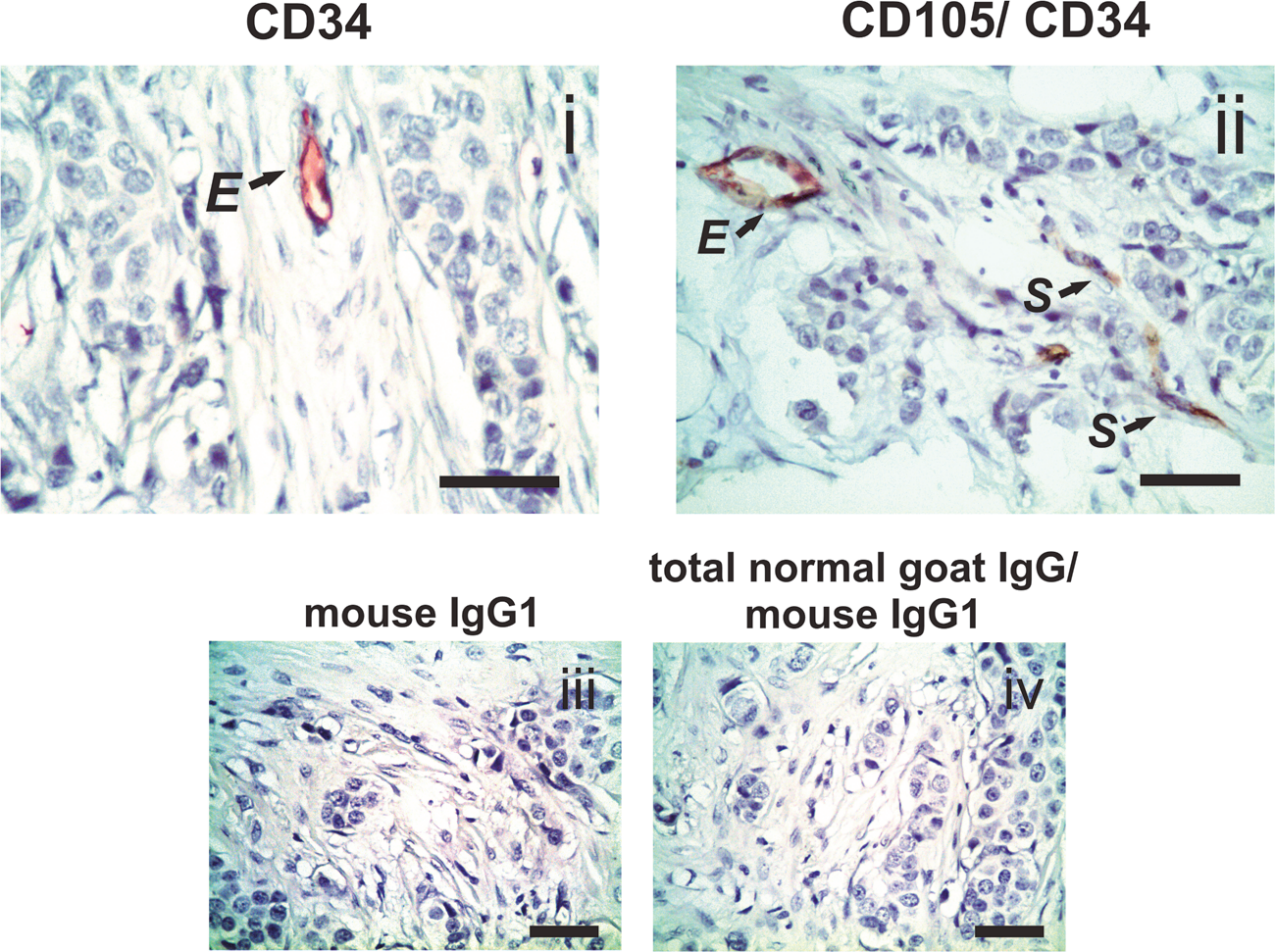
Expression of CD105 and CD34 in tumor stromal cells. **i** Single immunohistochemistry for CD34 (detected by red chromogen) shows a representative example of CD34-positive staining in the endothelial cells (E). **ii** Double immunohistochemistry for CD105 and CD34 (detected by brown and red chromogen, respectively) shows a representative example of co-staining of CD105 and CD34 in the endothelial cells (E) and CD105-positive staining only in evaluated stromal cells (S) of primary tumor tissue from a breast cancer patient. No staining was observed in the tissues when we incubated them with irrelevant mouse IgG1 (for CD34) or sequentially incubated with total normal goat IgG and irrelevant mouse IgG1 (for CD105/ CD34) as negative isotype controls (**iii and iv**; respectively). Nuclei were counterstained with hematoxylin (purple). Original magnification: 400×. The scale bars represent 50 μm.

Spindle-shaped stromal cells, not associated with vasculature, in BC tissues (n = 56) were found to be negative for CD34 expression (Fig. 2). In parallel, the double immunohistochemistry assay showed that the positive CD105-stromal cells with a spindle shape, not associated with vasculature, in BC tissues (n = 52) were negative for CD34 (Fig. 3). These findings demonstrate that these cells are not individual endothelial progenitor cells.

### Association between non-classical prognostic markers and classical prognostic markers, metastatic recurrence and local relapse of BCPs

CD105 expression in stromal cells with a spindle shape, not associated with the vasculature, was found to be significantly associated with tumor size in our patients (p = 0.0210). Thus, 46.15% (6/13) of the patients with tumors greater than 2 cm showed high CD105 expression, whereas 13.16% (5/38) of patients with tumors smaller than 2 cm showed high CD105 expression (Table 2). However, after the adjustment for multiple tests, this finding was considered not significant (Benjamini-Hochberg adjusted p = 0.2292). Moreover, high CD105 expression was associated with a significantly higher risk of developing metastasis (p = 0.0012; Benjamini-Hochberg adjusted p = 0.0224). Thereby, 63.64% (7/11) of samples from patients with metastasis had high CD105 expression, whereas 11.11% (4/36) of cases that remained metastasis-free had high CD105 expression (Table 2). Furthermore, when we considered the sites of metastases, we observed that 80.00% (4/5) of samples from patients with bone metastasis had high CD105 expression as well as 100.00% (3/3) of samples from patients with bone and visceral/non regional lymph node involvement. By contrast, no sample was obtained from patients with only visceral/non regional lymph node metastasis with high expression of this marker.

**Table 2:** Association of stromal cell expression of α-SMA, FSP, CD105 and CD146 with classical prognostic markers, metastatic recurrence and local relapse of 56 untreated early breast cancer patients.

*Characteristics*	*α-SMA*			*FSP*			*CD105*			*CD146*		
	*n*	*High expression*	*p (adjusted p)* *	*n*	*Positive*	*p (adjusted p)* *	*n*	*High expression*	*p (adjusted p)* *	*n*	*Positive*	*p (adjusted p)* *
***Age (years)***												
*< 50*	8	5 (62.50)	>0.9999	8	3 (37.50)	0.7032	8	2 (25.00)	>0.9999	8	2 (25.00)	0.4448
*≥ 50*	41	26 (63.41)	(>0.9999)	40	20 (50.00)	(0.9497)	39	9 (23.08)	(>0.9999)	43	19 (44.19)	(0.9249)
***Tumor size (cm)***												
*<2*	39	28 (71.79)	0.1913	38	22 (57.89)	0.2147	38	5 (13.16)	**0.0210**	41	16 (39.02)	>0.9999
*≥ 2*	14	7 (50.00)	(0.8084)	14	5 (35.71)	(0.8036)	13	6 (46.15)	(0.2292)	14	6 (42.86)	(>0.9999)
***Histological grade***												
*G1/G2*	30	21 (70.00)	0.5648	29	16 (55.17)	0.7804	30	5 (16.67)	0.3270	32	11 (34.37)	0.4055
*G3*	23	14 (60.87)	(0.9134)	23	11 (47.83)	(0.9736)	21	6 (28.57)	(0.8399)	23	11 (47.83)	(0.9319)
***HER2/neu status***												
*Negative*	32	20 (62.50)	0.5647	31	15 (48.39)	0.5822	28	6 (21.43)	>0.9999	32	12 (37.50)	0.7818
*Positive*	21	15 (71.43)	(0.9247)	21	12 (57.14)	(0.8868)	23	5 (21.74)	(>0.9999)	23	10 (43.48)	(0.9571)
***ER status***												
*Negative*	15	10 (66.67)	>0.9999	15	7 (46.67)	0.7619	13	3 (23.08)	>0.9999	15	8 (53.33)	0.2396
*Positive*	38	25 (65.79)	(>0.9999)	37	20 (54.05)	(0.9597)	38	8 (21.05)	(>0.9999)	40	14 (35.00)	(0.8483)
***PR status***												
*Negative*	14	8 (57.14)	0.5147	14	6 (42.86)	0.5364	12	2 (16.67)	0.7158	14	8 (57.14)	0.2059
*Positive*	39	27 (69.23)	(0.9496)	38	21 (55.26)	(0.9009)	39	9 (23.08)	(0.9472)	41	14 (34.15)	(0.8174)
***Regional lymph nodes***												
*Negative*	38	28 (73.68)	0.0524	37	22 (59.46)	0.2086	36	5 (13.89)	0.1180	40	15 (37.50)	0.7582
*Positive*	14	6 (42.86)	(0.4038)	14	5 (35.71)	(0.8037)	14	5 (35.71)	(0.6721)	14	6 (42.86)	(0.9643)
***Metastatic recurrence***												
*Negative*	37	25 (67.57)	0.4856	37	19 (51.35)	0.6180	36	4 (11.11)	**0.0012**	39	14 (35.90)	0.3109
*Positive*	11	6 (54.55)	(0.9495)	11	4 (36.36)	(0.9096)	11	7 (63.64)	**(0.0224)**	11	6 (54.55)	(0.8485)
***Local relapse***												
*Negative*	43	28 (65.12)	>0.9999	44	21 (47.73)	>0.9999	42	9 (21.43)	0.5776	45	18 (40.00)	>0.9999
*Positive*	5	3 (60.00)	(>0.9999)	4	2 (50.00)	(>0.9999)	5	2 (40.00)	(0.8902)	5	2 (40.00)	(>0.9999)

HER2: Human epidermal growth factor receptor 2; ER: Estrogen receptor; PR: Progesterone receptor; α-SMA: α-smooth muscle actin; FSP: Fibroblast surface protein, CD105: Endoglin; CD146: Melanoma cell adhesion molecule.

*The association between variables was performed using the Fisher Exact-test. Multiple testing adjusted p-values were computed using the Benjamini-Hochberg correction

**Fig 4:**
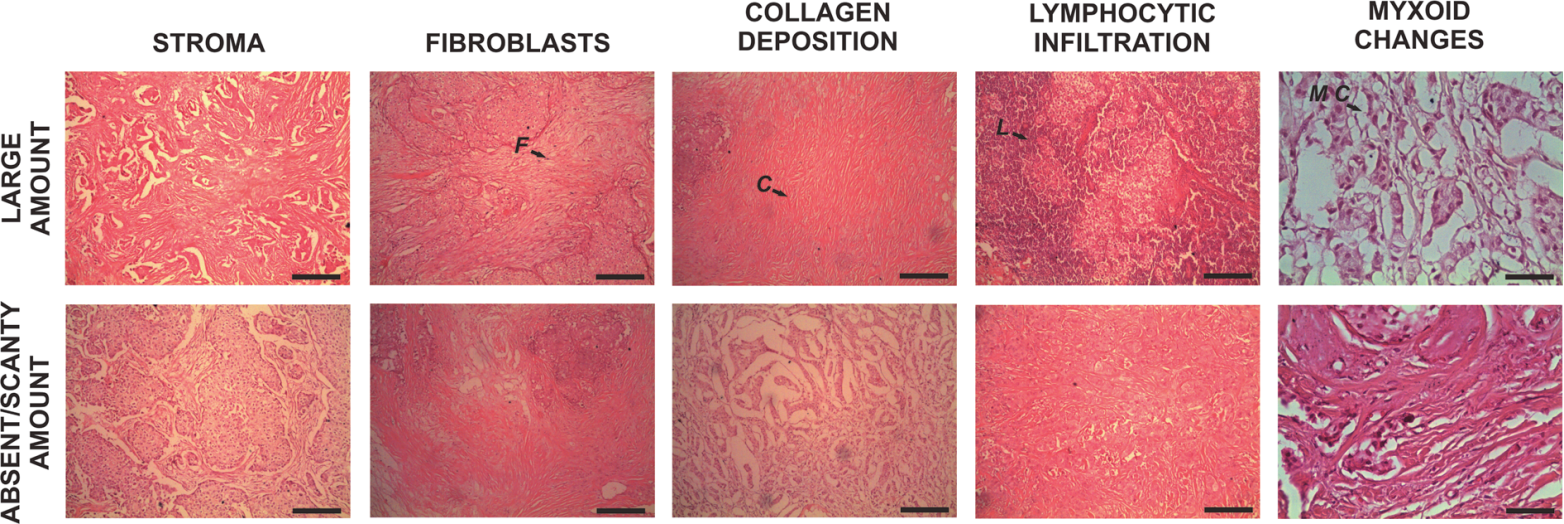
Histological features of primary breast tumor stroma as determined by hematoxylin and eosin staining. This picture is an example of samples with a large amount and absent/scanty amount of stroma, fibroblasts, collagen deposition, lymphocytic infiltration and myxoid changes. The arrows show tumor fibroblasts (F), collagen deposition (C), lymphocytic infiltration (L) and myxoid changes (M C). Original magnification: 100×. The scale bars represent 200 μm.

In addition, we found that the quantity of intra-tumor stroma was significantly associated with size and histological grade of the tumors in our patients (p = 0.0045 and 0.0120, respectively). So, 67.57% (25/37) of patients with tumors smaller than 2 cm were stroma-high (>50%), whereas 21.43% (3/14) patients with tumors greater than 2 cm were stroma-high. Additionally, 71.43% (20/28) of samples with G1/G2 were stroma-high, whereas 34.78% (8/23) of samples with poorly differentiated tumors (G3) were stroma-high (Table 3 and Fig. 4). However, after the Benjamini-Hochberg correction, these finding were considered not significant (Benjamini-Hochberg adjusted p = 0.0737 and 0.1747, respectively).

**Table 3:** Association of stromal histological features with classical prognostic markers, metastatic recurrence and local relapse of 56 untreated early breast cancer patients.

*Characteristics*	*n*	*Intra-tumor stroma*		*Fibroblasts*		*Collagen deposition*		*Lymphocytic infiltration*		*Myxoid changes*	
		*> 50*	*p (adjusted p)* *	*Large amount*	*p (adjusted p)* *	*Large amount*	*p (adjusted p)* *	*Large amount*	*p (adjusted p)* *	*Large amount*	*p (adjusted p)* *
***Age (years)***											
*< 50*	8	4 (50.00)	>0.9999	4 (50.00)	>0.9999	4 (50.00)	0.1868	1 (12.50)	0.6887	8 (100.00)	0.1767
*≥ 50*	40	22 (55.00)	(>0.9999)	18 (45.00)	(>0.9999)	31 (77.50)	(0.8739)	8 (20.00)	(0.9598)	30 (75.00)	(0.8573)
***Tumor size (cm)***											
*<2*	37	25 (67.57)	**0.0045**	20 (54.05)	0.3487	28 (75.68)	>0.9999	4 (10.81)	0.0519	27 (72.97)	**0.0450**
*≥ 2*	14	3 (21.43)	(0.0737)	5 (35.71)	(0.8381)	10 (71.43)	(>0.9999)	5 (35.71)	(0.4533)	14 (100.00)	(0.4211)
***Histological grade***											
*G1/G2*	28	20 (71.43)	**0.0120**	13 (46.43)	0.7812	21 (75.00)	>0.9999	3 (10.71)	0.2682	18 (64.28)	**0.0011**
*G3*	23	8 (34.78)	(0.1747)	12 (52.17)	(0.9654)	17 (73.91)	(>0.9999)	6 (26.09)	(0.8569)	23 (100.00)	**(0.0240)**
***HER2/neu status***											
*Negative*	30	17 (56.67)	0.7828	16 (53.33)	0.5725	21 (70.00)	0.5180	4 (13.33)	0.4601	22 (73.33)	0.1666
*Positive*	21	11 (52.38)	(0.9495)	9 (42.86)	(0.8928)	17 (80.95)	(0.9425)	5 (23.81)	(0.9273)	19 (90.48)	(0.9093)
***ER status***											
*Negative*	14	9 (64.29)	0.5327	5 (35.71)	0.3487	9 (64.29)	0.4723	4 (28.57)	0.2362	11 (78.57)	>0.9999
*Positive*	37	19 (51.35)	(0.9182)	20 (54.05)	(0.8381)	29 (78.38)	(0.9374)	5 (13.51)	(0.8595)	30 (81.08)	(>0.9999)
***PR status***											
*Negative*	13	7 (53.85)	>0.9999	5 (38.46)	0.5230	8 (61.54)	0.2743	3 (23.08)	0.6764	10 (76.92)	>0.9999
*Positive*	38	21 (55.26)	(>0.9999)	20 (52.63)	(0.9135)	30 (78.95)	(0.8356)	6 (15.79)	(0.9528)	31 (81.58)	(>0.9999)
***Regional lymph nodes***											
*Negative*	37	22 (59.46)	0.5204	18 (48.65)	>0.9999	28 (75.68)	0.7193	7 (18.92)	>0.9999	29 (78.38)	0.7134
*Positive*	13	6 (46.15)	(0.9337)	6 (46.15)	(>0.9999)	9 (69.23)	(0.9423)	2 (15.38)	(>0.9999)	11 (84.62)	(0.9536)
***Metastatic recurrence***											
*Negative*	36	19 (52.78)	0.7310	20 (55.56)	0.3177	25 (69.44)	0.2440	6 (16.67)	0.6631	28 (77.78)	>0.9999
*Positive*	11	7 (63.64)	(0.9481)	4 (36.36)	(0.8324)	10 (90.91)	(0.8196)	3 (27.27)	(0.9442)	9 (81.82)	(>0.9999)
***Local relapse***											
*Negative*	42	22 (52.38)	0.3623	23 (54.76)	0.1882	31 (73.81)	>0.9999	7 (16.67)	0.2397	34 (80.95)	0.5694
*Positive*	5	4 (80.00)	(0.8475)	1 (20.00)	(0.8501)	4 (80.00)	(>0.9999)	2 (40.00)	(0.8263)	3 (60.00)	(0.8987)

HER2: Human epidermal growth factor receptor 2; ER: Estrogen receptor; PR: Progesterone receptor.

*The association between variables was performed using the Fisher Exact-test. Multiple testing adjusted p-values were computed using the Benjamini-Hochberg correction

Regarding myxoid changes, we observed that they were significantly associated with size and histological grade of tumors in our patients (p = 0.0450 and 0.0011, respectively). As a result, 100.00% (14/14) of patients with tumors greater than 2 cm had a large amount of myxoid changes, whereas 72.97% (27/37) of patients with tumors smaller than 2 cm had a large amount of myxoid changes. Additionally, 100% (23/23) of samples with poorly differentiated tumors had a large amount of myxoid changes, whereas 64.28% (18/28) of samples with G1/G2 had a large amount of myxoid changes (Table 3 and Fig. 4). After the adjustment for multiple tests, only the association between myxoid changes and tumor histological grade remains significant (Benjamini-Hochberg adjusted p = 0.0240).

Finally, we found no other significant association between non-classical prognostic markers and classical prognostic markers, metastatic recurrence and local relapse in our BCPs (Tables 2 and 3).

### Association between non-classical prognostic markers and disease-free survival, metastasis-free survival and overall survival of BCPs

**Fig 5:**
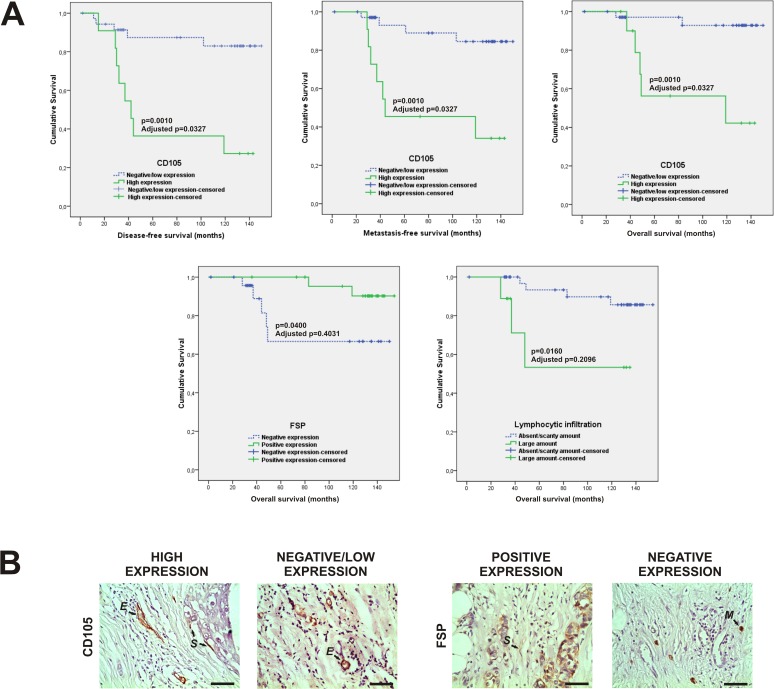
Correlation of CD105 and FSP expression, as well as lymphocytic infiltration quantity with disease-free survival, metastasis-free survival and overall survival of 56 untreated early breast cancer patients. **A** Kaplan-Meier curves show that high stromal cell expression of CD105 is associated with a shorter disease-free survival, metastasis-free survival and overall survival of our breast cancer patients. The difference is statistically significant [p = 0.0010 in all of cases by log-rank (Mantel-Cox)-test]. The adjusted p-value is 0.0327 (Benjamini-Hochberg correction) in all of cases. Moreover, positive FSP expression is significantly associated with a greater overall survival of these patients [p = 0.0400 by log-rank (Mantel-Cox) test]. Furthermore, a large amount of lymphocytic infiltration is significantly associated with a shorter overall survival of these patients [p = 0.0160, log-rank (Mantel-Cox) test]. After the Benjamini-Hochberg adjustment, these last associations were considered not significant (adjusted p = 0.4031 and 0.2096, respectively). **B** Photographs show a representative immunohistochemistry staining for tumor samples with high and negative/low stromal expression of CD105, as well as tumor samples with positive and negative FSP expression. The arrows show positive staining of evaluated stromal cells (S), endothelial cells (E) and tissue associated-macrophages (M) used as internal positive controls as previously described. Original magnification: 400×. The scale bars represent 50 μm.

Kaplan-Meier curve analysis indicated that CD105 expression was significantly associated with disease-free survival, metastasis-free survival and overall survival of our BCPs. Thus, patients with high CD105 expression showed a shorter disease-free survival, metastasis-free survival and overall survival than patients with negative/low CD105 expression (p = 0.0010; Benjamini-Hochberg adjusted p = 0.0327; in all of these cases) (Table 4 and Fig. 5).

**Table 4:** Comparing of the disease-free survival, metastasis-free survival and overall survival between untreated early breast cancer patients that presented high expression and those with negative/low expression of CD105.

*Clinical data*	*High expression* *	*Negative/low expression* *
*Disease-free survival*	70.64±15.40 (**a**)	132.01±7.62 (**a**)
*Metastasis-free survival*	81.73±15.45 (**b**)	136.11±6.66 (**b**)
*Overall survival*	96.62±15.51 (**c**)	143.58±4.42 (**c**)

* The data are expressed in months as mean±standard error. The p-value of **a**, **b** and **c** is 0.0010 [Kaplan-Meier method and analyzed by log-rank (Mantel-Cox)-test]. The adjusted p-value of **a**, **b** and **c** is 0.0327 (Benjamini-Hochberg correction)

In addition, univariate survival analysis showed a significant correlation of FSP expression with overall survival. Therefore, patients with positive FSP expression showed a greater overall survival than patients with negative FSP expression [data expressed in months as the mean±standard error; log-rank (Mantel-Cox) test]: 148.86±3.64 vs. 114.10±13.36, respectively; p = 0.0400 (Fig. 5). Additionally, we observed that the quantity of lymphocytic infiltration was significantly associated with the overall survival. So, patients with a large amount of lymphocytic infiltration showed a shorter overall survival than those with an absent or a scanty amount of lymphocytic infiltration [data expressed in months as mean±standard error; log-rank (Mantel-Cox) test]: 90.22±19.50 vs. 142.86±5.48, respectively, p = 0.0160 (Fig. 5). After the Benjamini-Hochberg correction, both associations were considered not significant (Benjamini-Hochberg adjusted p = 0.4031 and 0.2096, respectively).

Finally, no other significant association was found between non-classical prognostic markers and disease-free survival, metastasis-free survival and overall survival of patients (data not shown).

### Association between classical prognostic markers and disease-free survival, metastasis-free survival and overall survival of BCPs

Univariate survival analysis showed that tumor size was significantly associated with metastasis-free survival in our patients. Thus, patients with tumors greater than 2 cm had early metastatic recurrence [p = 0.0200 by log-rank (Mantel-Cox) test]. However, the Benjamini-Hochberg adjusted p value was 0.2382. Furthermore, no other significant association was found between classical prognostic markers and disease-free survival, metastasis-free survival and overall survival of patients (data not shown).

### Multivariate analysis

Multivariate analysis incorporating the significant variables without adjustment for multiple tests, demonstrated that CD105 expression was an independent predictive indicator for metastasis-free survival and overall survival of our BCPs (p = 0.0040 and 0.0130, respectively) (Table 5).

**Table 5:** Multivariate analysis of metastasis-free survival and overall survival in 56 untreated early breast cancer patients.

*Events*	*Characteristics*	*RR*	*95% C*.*I*.	*p* *
***Metastasis- free survival***	***CD105***			
	*High expression*	6.44	1.79–23.08	**0.0040**
	*Negative/low expression*	1	-	
	***Tumor size (cm)***			
	*≥ 2*	3.42	0.98–11.92	0.0530
	*<2*	1	-	
***Overall survival***	***FSP***			
	*Positive expression*	1	-	0.2590
	*Negative expression*	0.36	0.06–2.13	
	***CD105***			
	*High expression*	9.56	1.61–56.75	**0.0130**
	*Negative/low expression*	1	-	
	***Lymphocytic infiltration***			
	*Large amount*	5.19	0.94–28.60	0.0590
	*Absent/scanty amount*	1	-	

FSP: Fibroblast surface protein, CD105: Endoglin; C.I.: Confidence interval; RR: Relative risk.

*The Cox Proportional Hazards Model was applied to the multivariate survival analysis

## Discussion

Emerging evidence indicates that we need to consider carcinogenesis and tumor progression not as cell autonomous, but rather as a disease involving complex multicellular interactions within a newly formed tissue: the cancer tissue [32]. Although evidence suggests that the tumor-related stroma influences tumor behavior, no clinically applicable prognostic marker has emerged.

First, we evaluated the relationship between the intra-tumor stroma and clinicopathological data in our BCPs. The high quantity of the intra-tumor stroma was shown to be significantly associated with favorable classical prognostic markers such as size < 2 cm and low histological grade of the breast tumors. However, after the Benjamini-Hochberg correction, these finding were considered not significant. Meanwhile, Kruijf EM et al. [27] identified the tumor-stroma ratio as an independent prognostic factor in invasive BC and observed a shorter relapse-free period and overall survival in patients with stroma-rich tumors than in those with stroma-poor tumors. In contrast, Ahn et al. [33] did not find significant association between quantity of stroma and clinicopathological data of patients. Based on conflicting reports regarding the quantification of intra-tumor stroma, we not only examined its quantitative nature but also its qualitative nature.

In agreement with other authors, our data showed that the stromal cell expression of α-SMA and FSP was different between women with breast carcinoma and those with non-malignant breast tissue [34, 35], suggesting that these typical markers of CAFs could be useful in predicting the behavior of a patient's tumor. In this way, although there is a significant body of literature that demonstrates the role of FSP as a marker for BC progression [35, 36], the focus of these studies has been its expression in cancer cells. Our results showed that positive FSP expression was significantly associated with prolonged overall survival, suggesting that FSP-expressing CAFs are related to a favorable prognosis of early BCPs. However, this finding was not significant with adjustment for multiple tests.

We also observed that CD34-negative stromal cells with a spindle shape, not associated with the vasculature, in BC tissue expressed CD105 but we did not observe its expression in non-malignant breast tissue. Furthermore, we found that high CD105 expression in this stromal cell type was significantly associated with tumor size ≥ 2 cm. However, after the Benjamini-Hochberg correction, this result was considered not significant. Simultaneously, we demonstrated that high CD105 expression was significantly associated with a higher risk of developing metastasis and with a shorter disease-free survival, metastasis-free survival and overall survival of our patients. Moreover, after the adjustment for multiple tests, these last associations remain significant. Our results could be related to those of Li C et al. [37], who reported that the plasma levels of CD105 were significantly increased in patients with early-stage BC who developed distant metastasis compared with those in disease-free patients. In addition, if we consider the sites of metastases, high CD105 expression may be a predictive marker for the occurrence of skeletal metastasis in our BCPs. Indeed, the literature supports that BC relapses from bone marrow years after remission, suggesting a preferential niche in the hematopoietic microenvironment for circulating tumor cells [38]. Although there is no doubt that stromal CD105 expression has important prognostic implications, more efforts are required to determine if this expression is bone metastasis specific.

Concerning CD146 expression, our data indicated that malignant and non-malignant breast tissue expressed this marker, but no significant difference was found. Because we quantified the stromal cell expression of CD146 only in the tumor stroma that was not associated with the vasculature, it is possible that their expression was reduced or lacking as a consequence of the low oxygen levels in the reactive stromal compartment [39].

In reference to lymphocytic infiltration, our results only without Benjamini-Hochberg adjustment indicated that the high quantity of this infiltration was significantly associated with a shorter overall survival of our BCPs, supporting the belief that lymphocytic infiltration could be associated with poor prognosis as reported in previous studies [40–42]. By contrast, Mohammed ZM and et al. [43] reported that tumor lymphocytic infiltration was independently associated with improved overall survival of BCPs. Further study to characterize the type of lymphocytes present in the tumor stroma of our patients should be conducted in the future.

Another stromal signature are the myxoid changes, which consist of stromal reactions composed of amphophilic or slightly basophilic vacuolated material and are found among the collagen fibers. Tissues rich in hyaluronan lead to myxoid changes. Thus, strong evidence suggests that hyaluronan is associated with the invasive potential and poor outcome in BCPs [44, 45]. In accordance with previous results of other authors [28], we found that a large amount of stromal changes was associated with a high histological grade. We also observed that a large amount of myxoid changes was significantly associated with tumor size ≥ 2 cm in our patients. However, after the adjustment for multiple tests, only the first association previously described remains significant. Therefore, the presence of myxoid changes could help identify aggressive-tumors in early stage-BCPs.

In conclusion, this is the first demonstration of high CD105 expression in CD34-negative spindle-shaped stromal cells, not associated with the vasculature, as a possible independent marker of unfavorable prognosis in women with breast-infiltrative ductal carcinoma (I and II stages). Our study suggests that this novel result can be useful prognostic biomarker that would not only allow for more accurate selection of patients who would benefit from systemic therapy but could also lead to the development of new therapeutic strategies. We think that this finding can be considered a trigger for larger studies in this setting.
