# Correction: CD105 Expression on CD34-Negative Spindle-Shaped Stromal Cells of Primary Tumor Is an Unfavorable Prognostic Marker in Early Breast Cancer Patients

**DOI:** 10.1371/journal.pone.0126542

**Published:** 2015-04-15

**Authors:** The PLOS ONE Staff

During typesetting, errors were introduced into the names of the third, fifth, and eighth authors. The third author’s given name should be María de Luján, and the surname should be Calcagno. The fifth author’s given name should be Hernán, and the surname should be Garcia Rivello. The eighth author’s given name should be Valeria Beatriz, and the surname should be Fernández Vallone.

The correct citation is: Martinez LM, Labovsky V, Calcagno ML, Davies KM, Garcia Rivello H, Bianchi MS, et al. (2015) CD105 Expression on CD34-Negative Spindle-Shaped Stromal Cells of Primary Tumor Is an Unfavorable Prognostic Marker in Early Breast Cancer Patients. PLoS ONE 10(3): e0121421. doi:10.1371/journal.pone.0121421

The publisher apologizes for the errors.
